# The Effects of Methylene Blue on Autophagy and Apoptosis in MRI-Defined Normal Tissue, Ischemic Penumbra and Ischemic Core

**DOI:** 10.1371/journal.pone.0131929

**Published:** 2015-06-29

**Authors:** Zhao Jiang, Lora Talley Watts, Shiliang Huang, Qiang Shen, Pavel Rodriguez, Chunhua Chen, Changman Zhou, Timothy Q. Duong

**Affiliations:** 1 Department of Anatomy and Embryology, Peking University Health Science Center, Beijing, China; 2 Research Imaging Institute, University of Texas Health Science Center at San Antonio, San Antonio, Texas, United States of America; 3 Department of Anesthesiology and Critical Care, Perelman School of Medicine, University of Pennsylvania, Philadelphia, Pennsylvania, United States of America; Henry Ford Health System, UNITED STATES

## Abstract

Methylene blue (MB) USP, which has energy-enhancing and antioxidant properties, is currently used to treat methemoglobinemia and cyanide poisoning in humans. We recently showed that MB administration reduces infarct volume and behavioral deficits in rat models of ischemic stroke and traumatic brain injury. This study reports the underlying molecular mechanisms of MB neuroprotection following transient ischemic stroke in rats. Rats were subjected to transient (60-mins) ischemic stroke. Multimodal MRI during the acute phase and at 24hrs were used to define three regions of interest (ROIs): i) the perfusion-diffusion mismatch salvaged by reperfusion, ii) the perfusion-diffusion mismatch not salvaged by reperfusion, and iii) the ischemic core. The tissues from these ROIs were extracted for western blot analyses of autophagic and apoptotic markers. The major findings were: 1) MB treatment reduced infarct volume and behavioral deficits, 2) MB improved cerebral blood flow to the perfusion-diffusion mismatch tissue after reperfusion and minimized harmful hyperperfusion 24hrs after stroke, 3) MB inhibited apoptosis and enhanced autophagy in the perfusion-diffusion mismatch, 4) MB inhibited apoptotic signaling cascades (p53-Bax-Bcl2-Caspase3), and 5) MB enhanced autophagic signaling cascades (p53-AMPK-TSC2-mTOR). MB induced neuroprotection, at least in part, by enhancing autophagy and reducing apoptosis in the perfusion-diffusion mismatch tissue following ischemic stroke.

## Introduction

Stroke is a leading cause of death and long-term disability [1]. Following ischemic brain injury, the apoptotic cascade is activated within hours and culminates in progressive cell death into the expanding ischemic infarct through proteins in the Bcl-2 and caspase families [2]. Classically, cerebral ischemia increases p53 expression and downstream genes including Bax [3], a pro-apoptotic member of the Bcl-2 family of proteins [4]. Bax translocates to the mitochondria and induces the release of cytochrome c from the mitochondria into cytosol where it interacts with apoptotic protease activating factor 1 (Apaf-1) to activate caspase-9. The activation of caspase-9 in turn activates downstream caspases, such as caspase-3 that ultimately results in apoptotic cell death [4].

Autophagy, responsible for degradation and recycling of cellular components, is also activated following an ischemic insult [5]. Although the importance of autophagy in various biological and pathological processes is widely accepted [6], the role of autophagy in neuronal survival after acute cerebral ischemia is less well understood. There is evidence that autophagy contributes to the protective effect in cerebral ischemia [7] and is regulated by many different molecules, such as p53, AMP-activated protein kinase (AMPK), tuberous sclerosis complex 2 (TSC2) and the mammalian target of rapamycin (mTOR), through multiple signaling pathways [7–9]. Many drugs have attempted to target these (apoptotic and autophagic) molecular pathways to reduce ischemic brain injury.

Methylene blue (MB) USP is a FDA-grandfathered drug which is safely used to treat methemoglobinemia and cyanide poisoning in humans [10]. MB readily crosses the blood-brain barrier and has both energy-enhancing and antioxidant properties. MB acts as an electron cycler [10] which allows MB to redirect electrons to the mitochondrial electron transport chain (in the absence of oxygen), thereby sustaining or enhancing ATP production and promoting cell survival. In bypassing complex I-III activity to generate ATP, MB also reduces reactive oxygen species production from the mitochondrial electron transport chain [11], which has the potential to minimize ischemic and reperfusion injury. MB has recently been shown to reduce behavioral impairments in animal models of Parkinson's disease [10], Alzheimer’s disease [12,13], and to reduce behavioral impairments and lesion volume in traumatic brain injury [14]. We recently showed that MB administration reduced MRI-defined infarct volume and behavioral deficits in a rat model of ischemic stroke by middle cerebral artery occlusion (MCAO) [15].

In this study, we probed the underlying molecular mechanisms of neuroprotection of MB following transient ischemic stroke using western blot analysis of apoptotic and autophaphic cascades in different regions of interest (ROIs) defined by multimodal MRI. The combined use of diffusion and perfusion MRI [16,17] enabled the delineation of the “perfusion-diffusion mismatch” (which approximates the “ischemic penumbra”) and the ischemic core for histological analysis. The three ROIs were selected for Western blotting using multimodal MRI: i) the perfusion-diffusion mismatch (at-risk tissue) salvaged by reperfusion, ii) the perfusion-diffusion mismatch not salvaged by reperfusion, and iii) the ischemic core. Comparisons of western blot analysis were made with behavioral scores (neurological scores and foot fault analysis). Tissues from these ROIs were extracted for western blot analyses of autophagic and apoptotic markers. These findings shed light on the underlying molecular mechanisms involved in MB neuroprotection following transient ischemic stroke.

## Materials and Methods

### Experiment design

All experimental procedures were approved by the Institutional Animal Care and Use Committee at the University of Texas Health Science Center at San Antonio. Humane endpoints were used for these animals. The criteria were: at the end of selected time points, any animal experienced 20% weight loss, or any animal experiencing pain that is not controllable by analgesic. Additional criteria were used in consultation with a veterinarian as needed. The isoflurane-anesthetized animals will be euthanized by an overdose of pentobarbital followed by cervical dislocation.

Male Sprague-Dawley rats (280-320g, n = 25, Charles River Laboratories, Wilmington, MA) were randomized into three groups: i) sham surgery (n = 5), ii) transient (60mins) MCAO treated with 1mg/kg USP MB (American Regent, Shirley, NY) (n = 10, 5 rats were used for MRI and Western blot analysis, another group of 5 rats were used for behavioral testing), and iii) transient (60mins) MCAO treated with saline vehicle (n = 10, 5 rats were used for MRI and Western blot analysis, another group 5 rats were used for behavioral testing). Note that four animals, not included in the above groups, were excluded due to surgical complication or premature death and one animal was excluded due to the absence of stroke.

The animals were intubated and mechanically ventilated under isoflurane (2% in room air). The tail vein was catheterized. MCAO was achieved by inserting an intraluminal silicon rubber-coated filament (Doccol Corporation, Sharon, MA) through the external carotid artery in a retrograde fashion [15]. The animals were then secured in the supine position using a custom-made MRI-compatible stereotaxic headset. After the initial MRI measurement at 30mins after onset of stroke, vehicle (saline) or MB was infused via the tail-vein over 30mins using an MRI compatible pump (Harvard Apparatus). MB or vehicle was administered 30 mins after induction of MCAO but before reperfusion which was at 60 mins. It is now revised. Additional doses of vehicle or MB were given at 6hrs (1mg/kg, i.p.) and 15hrs (1mg/kg, i.p.) after MCAO. A previous study showed that the half life of MB is around 5.0–6.5 hours [18]. End-tidal CO_2_ (2.8–3.6%), rectal temperature (36.5–37.5°C), heart rate (340-450bpm) and arterial oxygenation saturation (92–97% for vehicle group and 93–98% for MB group) were recorded and maintained within normal physiological range during MRI.

### MRI Experiments

MRI was performed on a Bruker Biospec 7T/40cm scanner with a 76G/cm BGA12S gradient insert (Billerica, MA) using a custom-made surface coil for brain imaging and a neck coil for perfusion labeling [15]. Cerebral blood flow (CBF) was measured using the continuous arterial spin-labeling technique with gradient echo-planar imaging. Labeling duration was 2.7-s and post-labeling delay was 250ms. Apparent diffusion coefficient (ADC) was measured using spin-echo diffusion-weighted echo-planar imaging with gradients separately applied along the x, y, or z direction. Two b values of 4 and 1,200s/mm^2^ were used. Other MRI parameters were: single shot, matrix = 96x96 (reconstructed to 128x128), field of view = 25.6x25.6mm, seven 1.5mm thick slices, 90° flip angle, repetition time = 3s, echo time = 10.2ms for CBF and 30ms for ADC. ADC and CBF maps were acquired at 30 and 90mins post-occlusion and at 24hrs. T_2_ maps were also acquired on 24hrs using fast spin echo with four effective echo times (25, 40, 75 and 120ms), echo train length 8, and 8 signal averages.

Images from each rat at different time points were co-registered. ADC, CBF and T_2_ maps were calculated. The initial lesion volume was defined by the ADC lesion 30mins post stroke using the established threshold (0.53 × 10^−3^ mm^2^ /s) [19]. A threshold of 0.30 ml/g/min was used for measure CBF abnormal tissue volume. The perfusion-diffusion mismatch is the tissue with normal or close to normal ADC, but lower than 0.30 ml/g/min CBF. The final infarct volume was defined by T_2_ at 24hrs using the threshold of mean T_2_ value plus two times the standard deviation [15]. Edema-corrected infarct volume was calculated [15]. Three ROIs were chosen for western blot analysis and these ROIs were determined based on ADC and CBF MRI at 30mins and T_2_ MRI at 24hrs post-stroke. These ROIs were: ROI-A was the perfusion-diffusion mismatch area salvaged by reperfusion, ROI-B was the perfusion-diffusion mismatch area not salvaged by reperfusion, and ROI-C was the ischemic core with both perfusion and diffusion abnormalities. These definitions were applied at 30 mins after MCAO in order to define each region.

### Neurological deficits

The Garcia neurological scores [20] were analyzed in a blinded fashion at 24hrs. The foot-fault test was performed 1–3 days prior to MCAO and again 2 days post MCAO. The rat was placed on an elevated grid floor (size 18x11 in with grid openings of ~1.56 in^2^ and 1 in^2^) and video recorded for five minutes or until 50 steps were taken with one (non-affected) limb [14]. The percentage of foot faults for each limb were calculated as the number of right or left forelimb faults below the grid opening divided by the total number of steps taken.

### Western blot

The effect of MB on apoptotic and autophagic cascades was analyzed using western blot. Animals were sacrificed 24hrs post-occlusion. Coronal sections of the brain (7 slices, 1.5mm thick) were cut and each slice was matched to the MRI images. The brain tissue was separated based on the three ROIs defined by ADC and CBF maps at 30mins and T_2_ maps at 24hrs post-stroke. The collected tissue from each ROI was homogenized in ice-cold buffer (0.32M sucrose, 1mM EDTA, 1M Tris-HCL pH = 7.8 and dH_2_O plus 1 tablet Roche Protease Inhibitor). Aliquots of each fraction were used to determine the protein concentration of each sample using the bicinchoninic acid (BCA) protein assay (Thermo Scientific).

Protein samples (50**μ**g) were loaded onto a 12% polyacrylamide gel, electrophoresed, and transferred to a nitrocellulose membrane. The nitrocellulose membranes were then blocked, followed by incubation with the primary antibodies overnight at 4°C and then incubated with reciprocal secondary antibodies at room temperature for 2hrs. The primary antibodies (from Cell Signaling Technology unless otherwise specified) were: i) rabbit anti-caspase-3, ii) rabbit anti-Bax and Bcl-2, iii) rabbit anti-AMPK and β1/2, iv) rabbit anti-Phospho-AMPKα (Thr172), v) rabbit anti-Phospho-AMPKβ1 (Ser108), vi) rabbit anti-total/Phospho-mTOR (Ser2448), viii) rabbit anti-total/Phospho-TSC2 (Ser1387), ix) rabbit anti-LC3B (from MBL International), and x) rabbit anti-p53 (from MBL International). The dilutions were 1:1000 for primary antibodies and 1:2000 for secondary antibodies. Each western blot was analyzed using the C-DiGit Blot Scanner (LI-COR Biosciences) and Image Studio software (Version 3.1.4).

### Statistical analysis

Data were reported as mean ± SEM. The paired t-test was used to compare initial lesion volume and final infarct volume, and the unpaired t-test was utilized for comparisons between MB and control groups for the western blot results. *p*<0.05 was taken to be statistically significant.

## Results

### MRI outcomes

**Fig 1:**
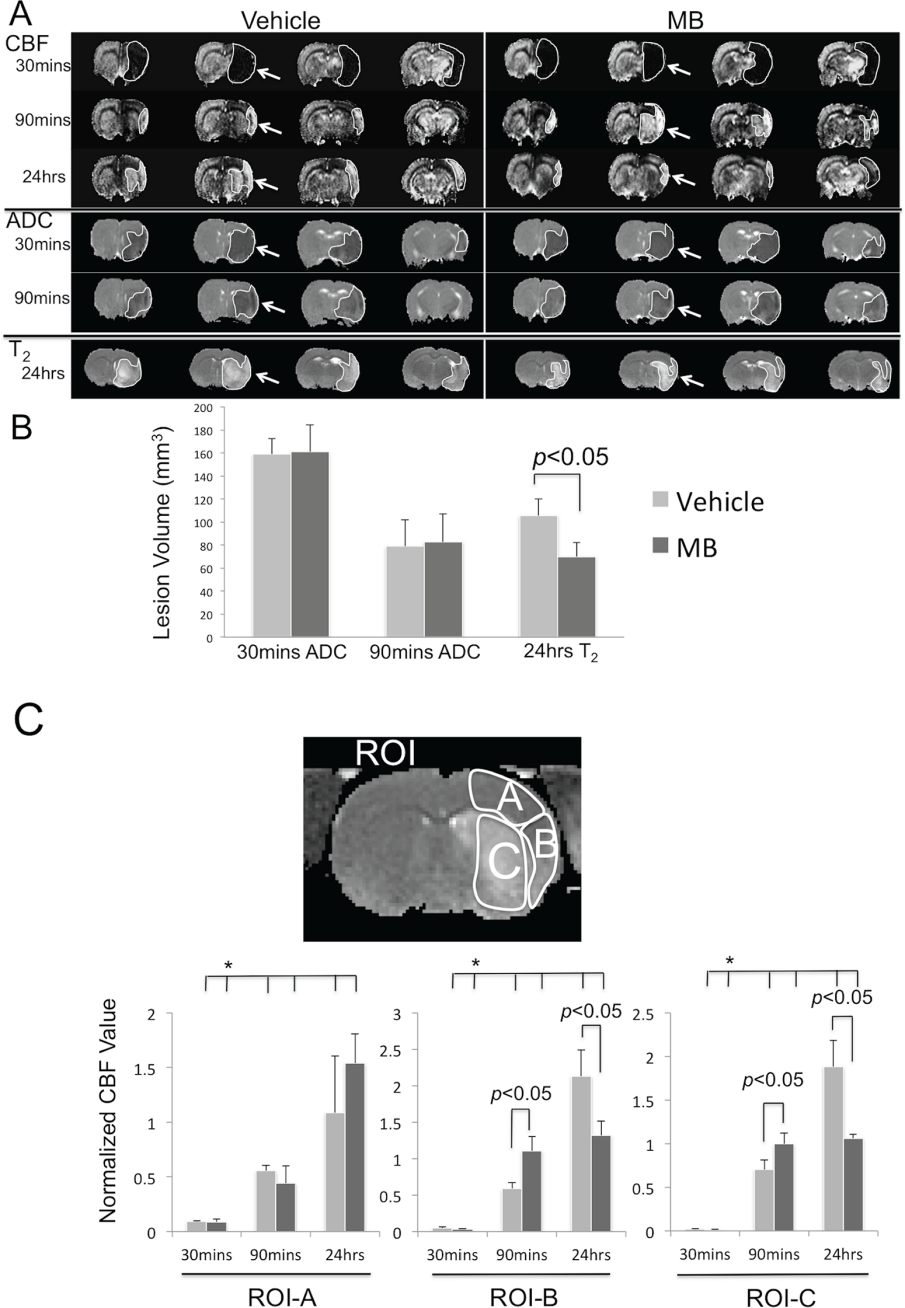
MB reduces infarct volume and CBF deficit. **(A)** Representative ADC, CBF and T2 maps, **(B)** lesion volumes, and **(C)** CBF value (normalized against the value at contralateral hemisphere) from three regions of interest (insets) at multiple time points post-occlusion of the vehicle and MB groups. The ROIs included: (ROI-A) the perfusion-diffusion mismatch area typically salvaged by reperfusion, (ROI-B) the perfusion-diffusion mismatch area typically not salvaged by reperfusion, and (ROI-C) the ischemic core. The ROIs and arrows delineated the regions of abnormalilties. Values are mean±SEM, n = 5 in each group. *p<0.05 indicates statistical difference from data at 30 mins.

Representative ADC, CBF and T_2_ maps at multiple time points (30mins, 90mins and 24hrs) post-occlusion from vehicle and MB rats are shown in Fig 1A. The ADC lesion volume at 30mins after MCAO was similar between the vehicle and MB groups, demonstrating a reproducible MCAO model. T_2_ infarct volume of the vehicle group was larger than that of the MB group 24hrs after MCAO.

The group-averaged lesion volumes were analyzed 30mins, 90mins and 24hrs after stroke (Fig 1B). The initial (30mins) ADC-defined lesion volumes before MB or vehicle administration were not statistically different from each other (161±24mm³ versus 159±13mm³, *p*>0.05). Lesion volumes at 90mins decreased in both MB and vehicle groups due to reperfusion. The final (24hrs) T_2_ infarct volume of the MB group was smaller than that of the vehicle group (70±12 versus 106±14mm³, *p*<0.05).

To investigate the effects of MB on CBF associated with cerebral ischemia, CBF of the vehicle and MB group were analyzed for three different ROIs: i) the perfusion-diffusion mismatch salvaged by reperfusion (ROI-A), ii) mismatch not salvaged by reperfusion (ROI-B), and iii) the ischemic core (ROI-C) (Fig 1C). The same ROIs were used for all time points. Before reperfusion (30mins), the CBF values of the three ROIs were not statistically different between the two groups. After reperfusion at 90mins, CBF values in both ROI-B and C (normalized CBF: 1.11±0.19 and 1.00±0.12, respectively) of the MB group were higher than those of the vehicle group (0.59±0.08 and 0.70±0.11, *p*<0.05 respectively), indicating that MB increased CBF after reperfusion. At 24hrs, the same ROIs in the MB group showed less hyperperfusion than that of the vehicle group.

### Behavioral outcomes

**Fig 2:**
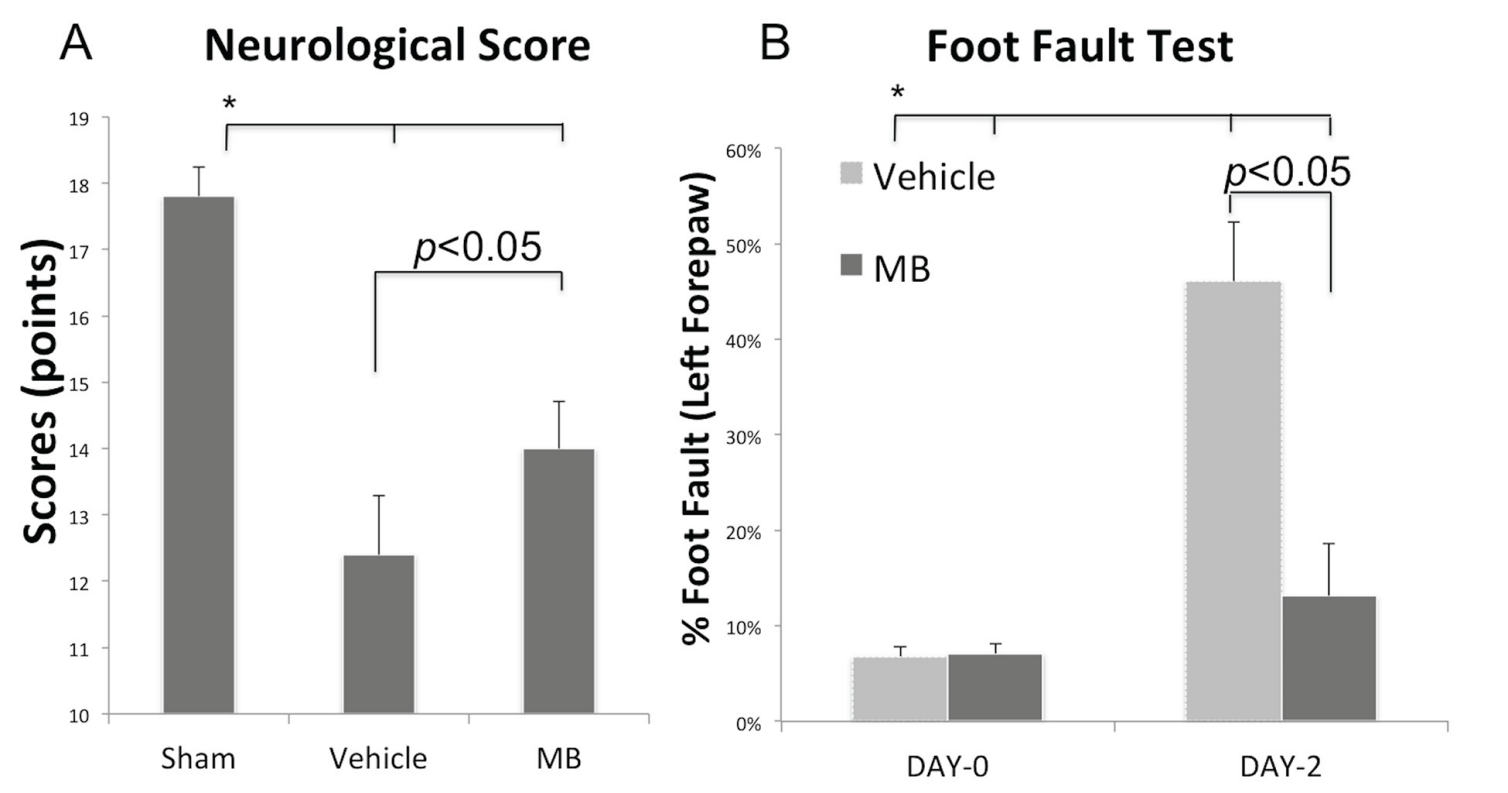
MB reduces behavioral deficits. **(A)** Neurological scores and **(B)** Foot fault scores of the MB group (n = 5) and the vehicle group (n = 5) 24hrs after MCAO. Values are mean±SEM.

Neurobehavioral scores were assessed using the Garcia scoring system at 24hrs (Fig 2A). Compared to sham, the vehicle group demonstrated poorer neurobehavioral scores as expected. By contrast, the MB group showed statistically significant improvement in neurobehavioral scores when compared to the vehicle group (14.0±0.7 vs. 12.4±0.8, *p*<0.05). Similarly, the foot fault scores of the affected forelimb in the MB group were significantly different from the vehicle group on 2 days post MCAO (0.13±0.03 vs. 0.46±0.06, *p*<0.05) but were not significantly different prior to stroke induction (Fig 2B).

### Effects of MB on key apoptotic and autophagic marker expression

**Fig 3:**
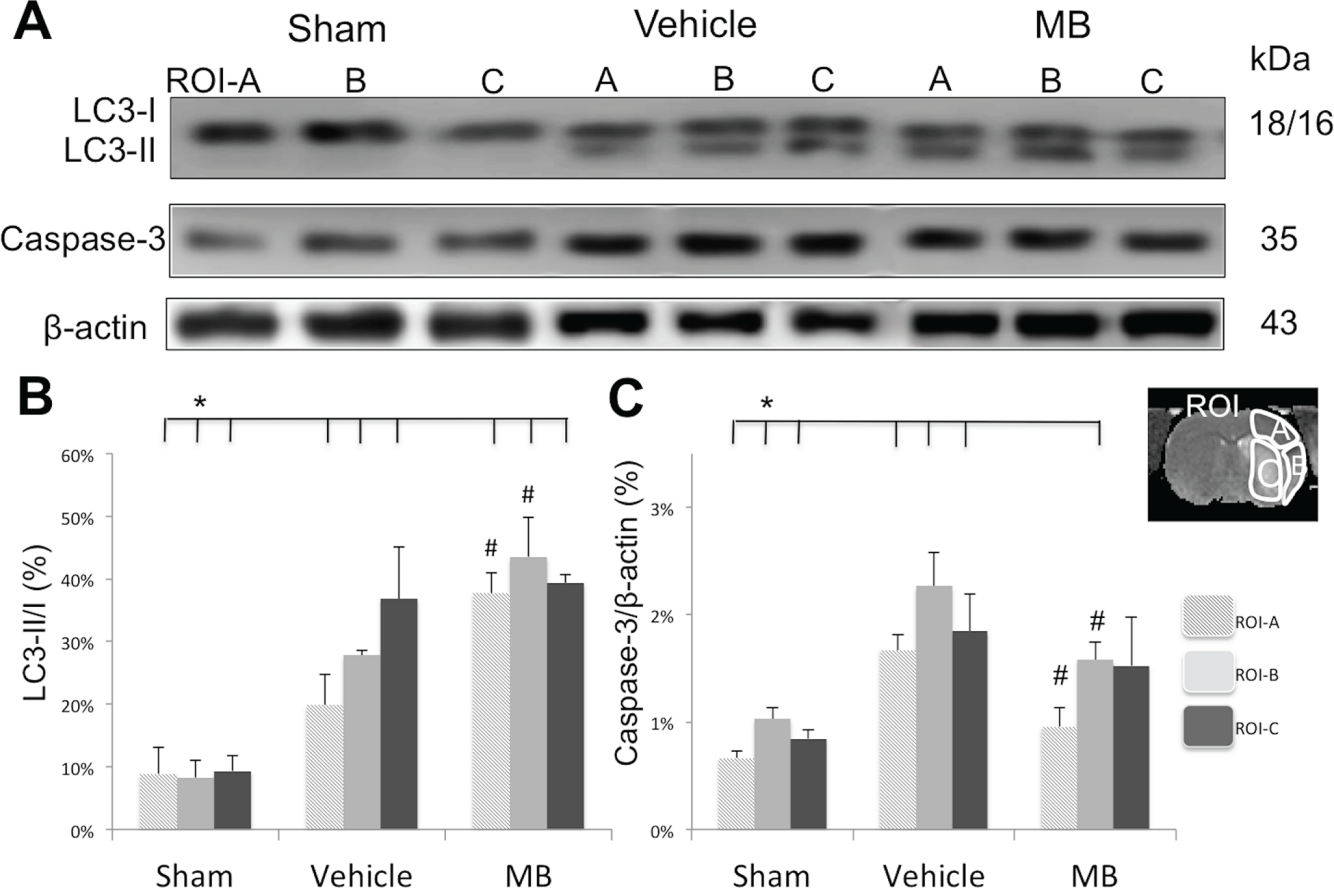
MB’s affects on autophagic and apoptotic markers 24hrs after stroke. Western blot analysis of autophagic marker LC3-II/I ratio, and apoptotic marker Caspase-3 in sham, vehicle and MB groups. The ratio of expression levels of LC3-II/I (B) were significantly increased in ROI-A and ROI-B of MB treated group compared to that of vehicle group (^#^ *p*<0.05). Caspase-3 expression level (C) was significantly increased in ROI-A and ROI-B of MB treated group compared to that of vehicle group (^#^ *p*<0.05). *p<0.05 indicates statistical difference from sham. Values are mean±SEM, n = 5 in each group.

Western blot analysis was performed to determine the ratio of expression of autophagy markers (LC3 I and II) and the expression of an apoptotic marker (caspase-3) 24hrs after stroke in the three ROIs (Fig 3). Compared with the sham group, both the vehicle and MB groups demonstrated significantly increased LC3-II/I ratios and reduced caspase-3 expression levels in the three ROIs at 24hrs. Compared with the vehicle group, MB significantly increased the ratio of LC3-II/I and reduced caspase-3 in ROI-A and B (*p*<0.05), but not in ROI-C, indicating that MB increased autophagy and inhibited apoptotic cell death in ROI-A and B, but not in ROI-C. In the ischemic core (ROI-C), both the LC3-II/I ratio and caspase-3 expression appeared to increase in the vehicle group. However, MB had no effect on these proteins.

### Effects of MB on downstream apoptotic pathway

**Fig 4:**
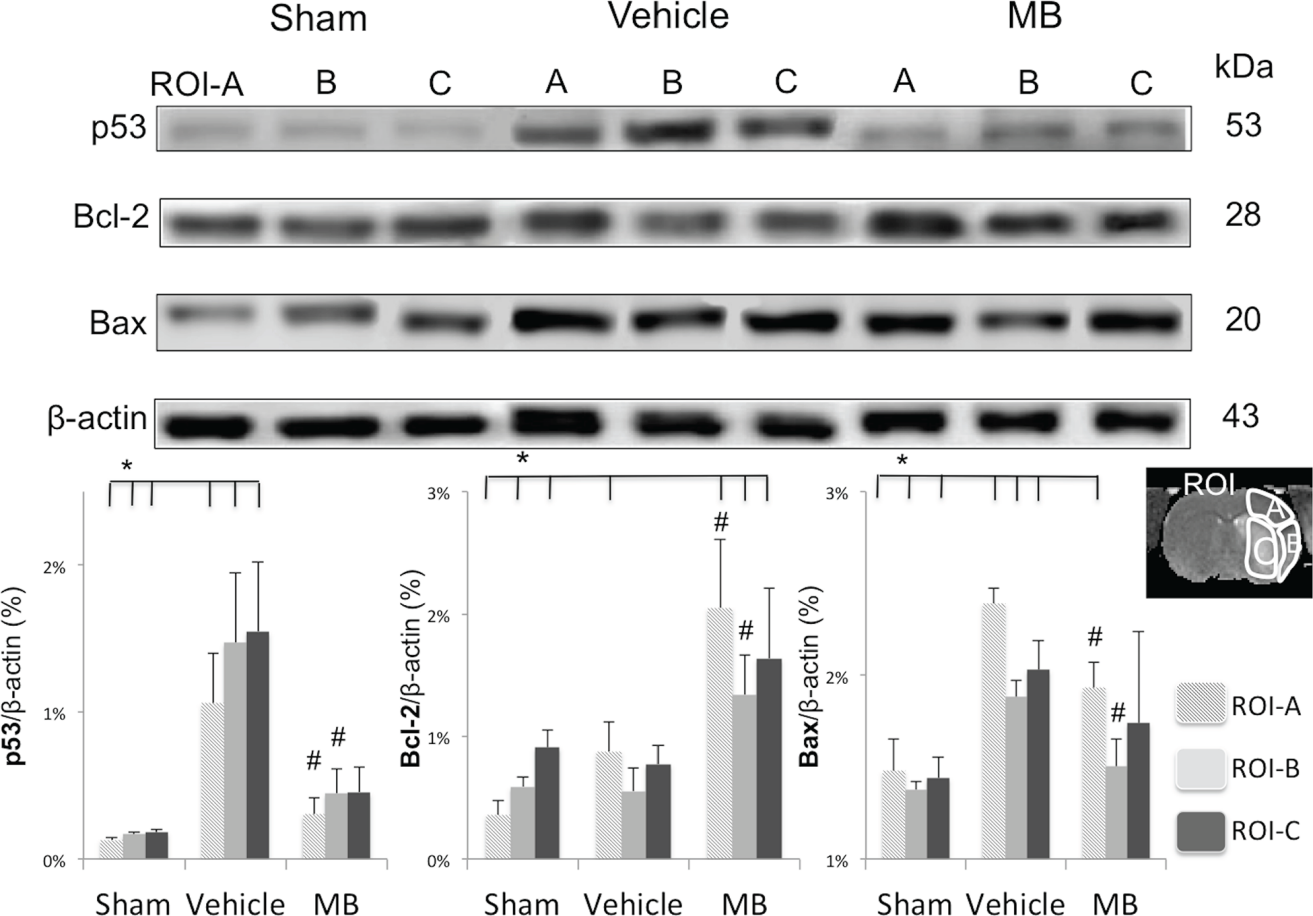
Western blots of p53, Bcl-2 and Bax 24hrs after stroke in the same ROI of the sham, vehicle and MB group. *p<0.05 indicates statistical difference from sham. ^#^ *p<*0.05 versus vehicle in same ROI. Values are mean±SEM, n = 5 in each group.

To further probe the effects of MB on the downstream apoptotic processes, p53, Bcl-2 and Bax protein expression levels were analyzed in the three ROIs (Fig 4). The group-averaged p53 expression level in all ROIs of the vehicle group increased significantly compared to the sham group. The MB group showed markedly reduced p53 protein levels in all three ROIs compared to the vehicle group (*p*<0.05). The MB group also showed reduced Bax expression in ROI-A and B but not in ROI-C compared to the vehicle group. MB also enhanced Bcl-2 expression in ROI-A and B but not in ROI-C compared to vehicle (*p*<0.05). Collectively, MB inhibited the molecular cascades associated with the classic apoptotic signaling pathway in the at-risk tissue but not in the ischemic core following ischemic stroke.

### Effects of MB on downstream autophagic pathway

**Fig 5:**
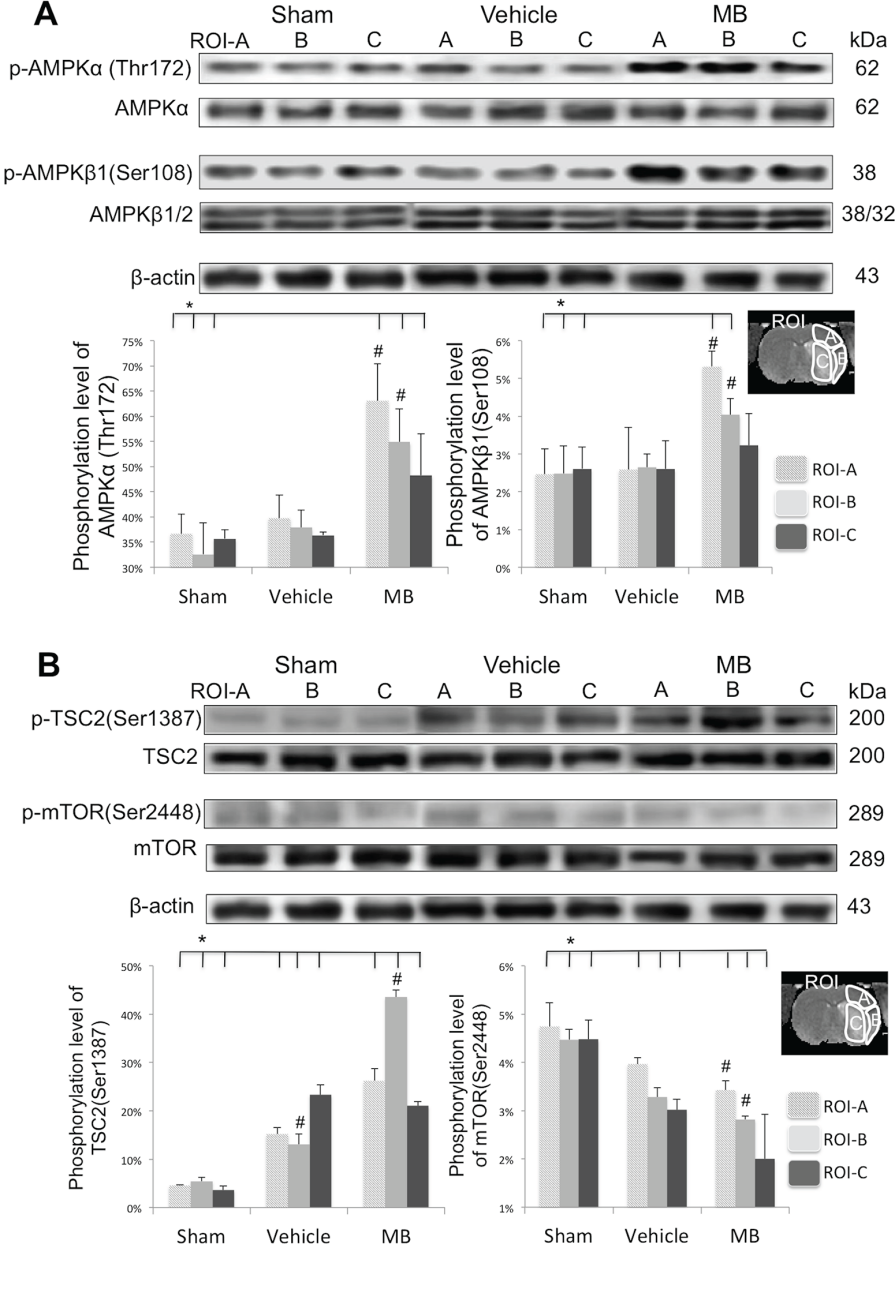
**(A)** Western blots of AMPK through the hyper-phosphorylated of AMPKα at Thr172 site and AMPKβ1 at Ser108 site 24hrs after stroke of vehicle and MB group. **(B)** Western blots of phosphorylation level of TSC2 at site Ser1387 and mTOR at site Ser2448 24hrs after stroke of vehicle and MB group. *p<0.05 indicates statistical difference from sham. ^#^ *p<*0.05 versus vehicle in same ROI. *Values* are mean±SEM, n = 5 in each group.

To further probe the effects of MB on the induction of autophagy, western blot analysis was used to determine whether there was a link between p53 expression and the phosphorylation level of AMPKα/β at specific sites for the three ROIs (Fig 5A). The MB group showed considerable hyper-phosphorylation of AMPKα at the Thr172 site in ROI-A and B but not in ROI-C compared to the sham and vehicle groups (*p*<0.05). Similarly, the MB group also demonstrated considerable hyper-phosphorylation of AMPKβ1 at the Ser108 site, compared to the sham and vehicle groups in ROI-A and B (*p*<0.05), but not in ROI-C.

The phosphorylation level of TSC2 (downstream protein of AMPK) at the Ser1387 site was also measured (Fig 5B). The group-averaged phosphorylation levels of TSC2 were significantly enhanced by MB in both ROI-A and B (*p*<0.05) but not ROI-C. Because TSC2 acted downstream of AMPK to inhibit mTOR [21], the phosphorylation level of mTOR at site Ser2448 was also measured. Compared to sham, vehicle-treated stroke rats demonstrated decreased phosphorylation of mTOR in ROI-A and B (but not ROI-C), and MB-treated stroke rats displayed a further reduction in the phosphorylation of mTOR in ROI-A and B but not in ROI-C.

**Fig 6:**
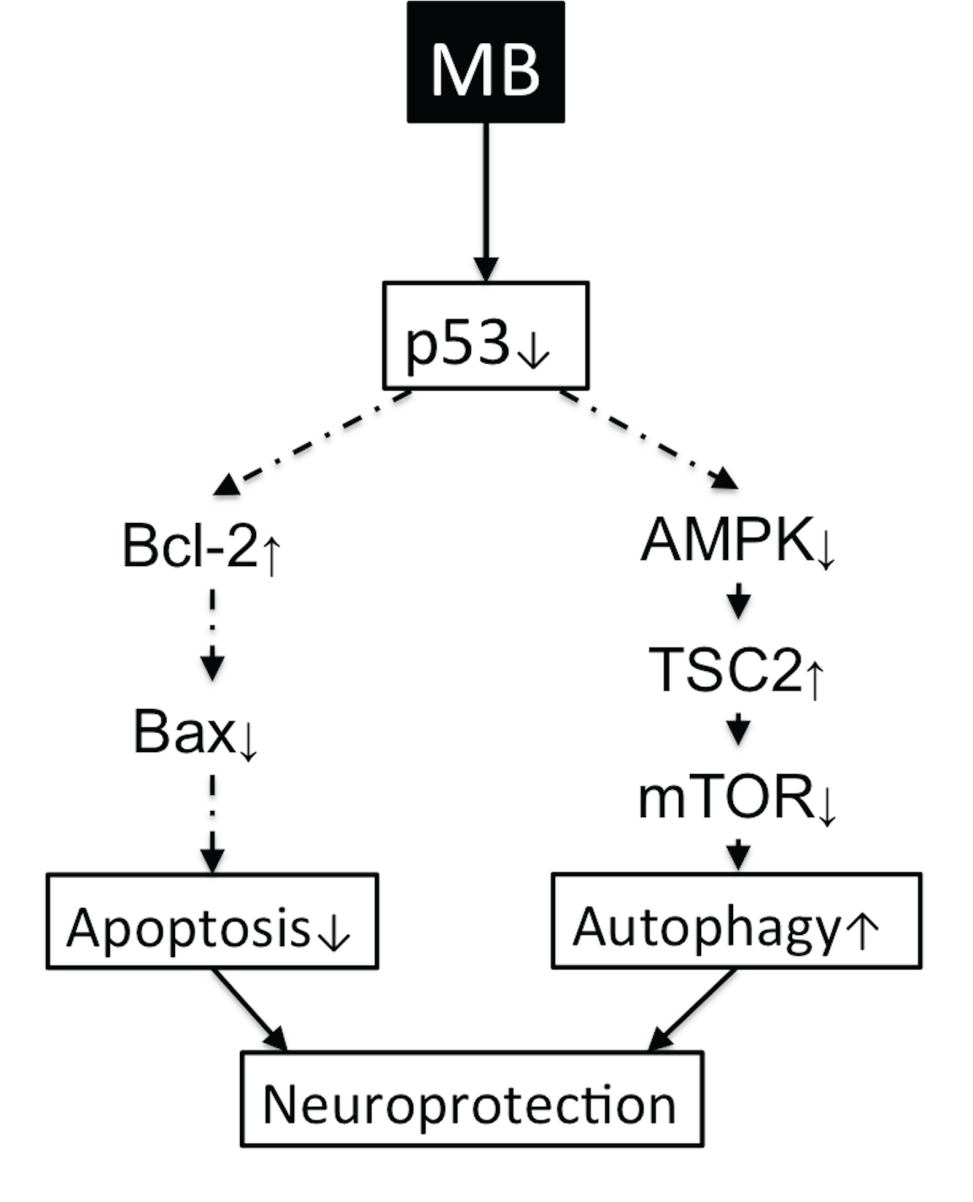
A schematic illustration of the effect of MB on the inhibition of apoptosis and induction of autophagy. MB induces neuroprotection in neurons following cerebral ischemia through both the apoptotic p53-Bcl-2-Bax signaling pathway and autophagic p53-AMPK-TSC2-mTOR signaling pathway. The arrows show the effect of MB on these pathways.

Fig 6 summarizes our findings of the effects of MB on autophagy and apoptosis. MB increased autophagic processes and inhibited apoptotic cell death processes in the at-risk tissue following ischemic stroke. This process involves the p53 pathway.

## Discussion

This study probed the underlying molecular mechanisms of MB neuroprotection following transient ischemic stroke in rats. The major findings are: 1) MB reduces infarct volume and behavioral deficits following transient ischemic stroke in rats, 2) MB improves CBF to at-risk tissue after reperfusion and minimizes harmful hyperperfusion 24hrs after MCAO, 3) MB inhibits apoptosis and enhances autophagy in the at-risk tissue but not within the ischemic core, 4) MB modulates the p53-Bax-Bcl2-caspase3 cascade, inhibiting apoptotic signaling pathways, 5) MB modulates p53-AMPK-TSC2-mTOR cascades, enhancing autophagic signaling pathways.

### The Advantage of MRI Application In Stroke Research

MRI provides clinically relevant information and is widely used to study preclinical and clinical stroke. Diffusion-weighted imaging (DWI) in which contrast is based on water apparent diffusion coefficient (ADC) is recognized as a useful imaging modality because of its ability to detect ischemic brain injury within minutes after onset [22]. Following hyperacute ischemic stroke, there is usually an ischemic core and a surrounding ischemic penumbra which has reduced CBF but preserved energy metabolism [23]. The ischemic penumbra is an important target for treatment. The combined use of ADC and CBF MRI allows delineation of normal, at risk (penumbra) and ischemic core tissues in the hyperacute phase [24,25]. Multimodal MRI was used in this study to guide the extraction of different tissue types for subsequent western blot analysis in individual animals. These tissue types were the perfusion-diffusion mismatch salvaged by reperfusion (ROI-A), the perfusion-diffusion mismatch not salvaged by reperfusion (ROI-B), and the ischemic core (ROI-C), which would not have been possible with terminal histological analysis [26,27].

In this study, the ischemic penumbra was approximated by the perfusion-diffusion mismatch and the thresholds used for the mismatch definition was based on previously established thresholds [19]. We are cognizant that these thresholds may not be universally applied to all conditions. Moreover, clinical trials using ‘mismatch’ as a selection criterion for thrombolysis has shown variable success [28–30] and its merit is still being debated [31,32]. Other techniques based on T2’ (quantitative T2* corrected with spin–spin effects, [33]), pH [34] and T2*-weighted imaging of oxygen challenge [35–37] are being developed to improve delineation of the ischemic penumbra. Nonetheless, the use of the perfusion-diffusion mismatch concept is useful in our studies. Our overall conclusion remains valid. Despite its limitation, the perfusion-diffusion mismatch concept remains useful to date clinically.”

### The Role of MB In Stroke-induced Apoptotic Cell Death

In the ischemic core, tissue necrosis dominates, whereas in the penumbra, programmed cell death pathways, such as apoptosis [2,3,38] and autophagy [5–7] dominate the progression of cell death.

Apoptosis can be induced by either cell surface death receptors or through mitochondrial cytochrome c release, and many molecules are closely involved in the regulation of each coordinated pathway. Caspase-3 activation, a common denominator for both apoptosis inducing pathways, has previously been shown to increase following ischemic brain injury [39]. Our results showed that MB inhibited caspase-3 expression in the penumbra ROI, but not in the core ROI. To further probe the effect of MB on apoptotic pathways, two members of the B-cell lymphoma class proteins were measured: Bcl-2 (anti-apoptotic) and Bax (pro-apoptotic). We found that MB significantly suppressed Bax expression and up-regulated Bcl-2 protein levels in the penumbra ROI, but not in the core ROI. Furthermore, we assessed the expression of p53, known to trigger the intrinsic apoptotic pathway, and found that MB also suppressed p53 expression in the penumbra ROI. These data suggest that MB neuroprotection acted through inhibition of p53 and Bax, and enhancement of pro-survival Bcl-2. MB inhibited apoptotic cell death by stabilization of the mitochondria, limiting permeability of the outer mitochondrial membrane and thus blocking the release of pro-apoptotic inducing molecules. Our findings are in agreement with previous studies where MB was found to stimulate several anti-apoptotic genes, and activated brain repair/regeneration mechanisms [40,41]. In short, our data are the first to demonstrate that MB inhibits the apoptotic p53-Bax-Bcl2-caspase3 pathway, suppressing apoptotic cell death in the penumbral ROIs, thereby preventing the transition into infarct and decreasing overall infarct volume.

MB has similar effects in both ROI-A and ROI-B (both ROIs were classified as perfusion-diffusion mismatch), whereas tissue within ROI A survived, but tissue within ROI B died. Fig 3C shows the expression of Caspase-3 in ROI-B of the MB group significantly deceased compare to the vehicle group. However, the expression of Caspase-3 in the ROI-B region of the MB group was still at the same level of the ROI-A region of the vehicle group. This indicates that even though MB treatment partially reduces the apoptotic process, the recovery was insufficient to rescue the tissue within ROI-B. Additionally, we also found a significant difference between the Sham and MB groups in the ROI-B region, but not in the ROI-A region, which indicates that the apoptotic level returned to normal in the ROI-A region.

### The Role of MB In Stroke-induced Autophagic Cell Death

Autophagy is a highly regulated process that breaks down organelles and macromolecules through lysosomal degradation and is essential for maintenance of intracellular homeostasis. Autophagy plays an important role in salvaging at-risk tissue in the penumbra during the acute phase, as well as in improving functional recovery in the chronic phase. Hyperactivation of autophagy, however, can lead to increased cell death. Autophagy is thus considered a double-edged sword.

Autophagosomes are formed during autophagy as a mechanism of delivering sequestered cellular debris to lysosomes for degradation. To date LC3, a mammalian homolog of the yeast Atg8, is the only reliable marker for autophagosomes as it is a specific constituent of the autophagosomal membrane [42]. LC3 begins as cytoplasmic LC3-I and is recruited to the autophagosome during autophagy, whereas LC3-II is subsequently generated by proteolysis and lipidation [43]. Tracking the conversion of LC3-I to LC3-II by immunoblotting provides a measure of autophagic activity [5]. Our results showed that the LC3-II to LC3-I ratio increased in all three ROIs in the vehicle and MB groups compared to the sham group. However, only the at-risk tissue showed increased LC3-II to LC3-1 ratio with MB compared to the vehicle group. Previous studies have demonstrated that chloroquine (known to block autophagy) inhibits MB-mediated neuroprotection [44]. In short, we concluded that the neuroprotective effect of MB is mediated by up-regulating autophagy only in the penumbra region, but not the ischemia core.

The regulation of autophagy is strongly connected to signaling pathways that promote cell proliferation and nutrient signaling (i.e., mTOR, p53/AMPK and PI3-K). Our data support the hypothesis that MB activates autophagy through the p53-AMPK-mTOR signaling pathway. The AMPKα (at Thr172 site) and AMPKβ1 (at Ser108 site) were found to be hyper-phosphorylated, consistent with a previous study that demonstrated MB-induced AMPK activation was accompanied by acetyl-CoA carboxylase promotion which is a downstream protein of AMPK (Xie et al., 2013). Moreover, we also found that mTOR (at site Ser2448) was hypo-phosphorylated in the mismatch tissues of the MB group. This finding is consistent with a previous study that reported that MB induces autophagy through inhibiting mTOR activation in neurodegenerative transgenic cell lines [45].

There are multiple regulatory factors that control mTOR activity that ultimately impacts the activation level of autophagy. Whether MB acts via the mTOR pathways however remains controversial [44,45]. We thus probed the molecular mechanisms of the downstream AMPK pathway. The AMPK pathway plays a key role in controlling cellular metabolism and energy homeostasis [7,21]. AMPK is known to phosphorylate and enhance TSC2, which inhibits the mTOR pathway, promoting autophagy [7,21]. We found that TSC2 was indeed hyper-phosphorylated at the Ser1387 site in MB-treated group, supporting the notion that MB acts via the AMPK and mTOR pathway in regulating autophagy. Furthermore, this phosphorylation was greater in ROI-A than ROI-B, indicative of the different extents of downstream autophagy induction between salvaged versus non-salvaged at-risk tissue. Taken together, we concluded that MB-induced autophagy is mediated by inhibition of p53, which subsequently activates AMPK causing inhibition of mTOR in the penumbral area.

Our data indicated that MB acts on apoptosis and autophagy through modulating p53 activity. p53-induced apoptotic cell death is known to occur via regulation of gene expression and promotion of mitochondrial membrane integrity [46]. During hypoxia, nuclear p53 reduces autophagy by suppressing the expression of BNIP3 [47]. In contrast, inhibition of cytoplasmic p53 can trigger autophagy [48]. Our data showed that MB likely acts to suppress both nuclear and cytoplasmic p53, inhibiting the apoptotic p53-Bax-Bcl2-caspase3 pathway while enhancing the autophagic p53-AMPK-TSC2-mTOR signaling pathway.

At the systemic level, MB has been shown to increase CBF, brain glucose and oxygen consumption under normoxic and hypoxic conditions [49], as well as enhance evoked responses due to forepaw stimulation [50]. We found that MB improved CBF to at-risk tissue post-reperfusion. Furthermore, we also found MB minimized harmful hyperperfusion 24hrs after MCAO, which has been previously shown to correlate with poor outcome in ischemic stroke [51]. MB has also been shown to decrease reactive oxygen species production in ischemia/reperfusion injury [52] and neuron cell death induced by oxidative stress [53]. The improvement of energy production and the reduction of oxygen free radicals likely contribute to the stabilization of the mitochondria and subsequent neuroprotection during transient ischemic stroke.

MB has an excellent safety profile and is used clinically to treat a number of indications, enabling speedy translation to clinical trials if it is proven efficacious in stroke animals. Improving understanding of the mechanisms underlying MB neuroprotection following stroke in vivo will help to better design MB clinical trials and MB treatment regimens for stroke patients in the future. A unique advantage of MB is that it could in principle be administered on site by emergency responders, which could potentially benefit a larger acute stroke patient population.

## Conclusion

This study demonstrated that MB-induced neuroprotection in ischemic stroke is mediated by enhancing autophagy and suppressing apoptosis. Multimodal MRI enabled the extraction of different tissue types for western blot analysis, and these tissue types included the perfusion-diffusion mismatch (at-risk tissue) salvaged by reperfusion, the perfusion-diffusion mismatch not salvaged by reperfusion, and the ischemic core. Our data showed that MB modulated p53 expression, which in turn modulated both the autophagic AMPK-TSC2-mTOR signaling pathway and the apoptotic Bax-Bcl2-caspase3 signaling pathway. The extent of autophagy and apoptosis differed among the different MRI-defined ischemic tissues, and between the vehicle and MB groups. This study shred lights on the underlying molecular mechanisms of MB neuroprotection in ischemic stroke.
